# Rapid Stencil Mask Fabrication Enabled One-Step Polymer-Free Graphene Patterning and Direct Transfer for Flexible Graphene Devices

**DOI:** 10.1038/srep24890

**Published:** 2016-04-27

**Authors:** Keong Yong, Ali Ashraf, Pilgyu Kang, SungWoo Nam

**Affiliations:** 1 Department of Mechanical Science and Engineering, University of Illinois at Urbana−Champaign, Urbana, Illinois 61801, United States; 2 Department of Materials Science and Engineering, University of Illinois at Urbana−Champaign, Urbana, Illinois 61801, United States

## Abstract

We report a one-step polymer-free approach to patterning graphene using a stencil mask and oxygen plasma reactive-ion etching, with a subsequent polymer-free direct transfer for flexible graphene devices. Our stencil mask is fabricated via a subtractive, laser cutting manufacturing technique, followed by lamination of stencil mask onto graphene grown on Cu foil for patterning. Subsequently, micro-sized graphene features of various shapes are patterned via reactive-ion etching. The integrity of our graphene after patterning is confirmed by Raman spectroscopy. We further demonstrate the rapid prototyping capability of a stretchable, crumpled graphene strain sensor and patterned graphene condensation channels for potential applications in sensing and heat transfer, respectively. We further demonstrate that the polymer-free approach for both patterning and transfer to flexible substrates allows the realization of cleaner graphene features as confirmed by water contact angle measurements. We believe that our new method promotes rapid, facile fabrication of cleaner graphene devices, and can be extended to other two dimensional materials in the future.

Graphene, a two-dimensional carbon allotrope, has received immense scientific and technological interest since its discovery^1^. Graphene’s combination of exceptional mechanical properties^2^, superior carrier mobility^3^, high thermal conductivity^4^, hydrophobicity^5^ and potentially low manufacturing cost^6^ has signified itself as a superior base material for next generation bioelectrical, electromechanical, optoelectronic, and thermal management applications^7^,^8^,^9^,^10^.

Significant progress has been made in the direct synthesis of large-area, uniform, high quality graphene films using chemical vapor deposition (CVD) with various precursors and catalyst substrates^11^,^12^,^13^. However, to date, the infrastructure requirements on post-synthesis processing (*e.g.*, patterning and transfer) for creating interconnects, transistor channels, or device terminals have slowed the implementation of graphene in a wider range of applications^14^,^15^. Existing methods to pattern graphene can be categorized into conventional photolithography^16^, soft lithography or transfer printing^17^,^18^, direct patterning using ion beam^19^, laser scribing^20^, ablation^21^, or hydrogen desorption^22^ after transfer to an appropriate substrate, and direct growth of patterned graphene^14^,^23^,^24^,^25^,^26^,^27^. However, despite the plethora of these aforementioned methods, they have limitations that have prevented low-cost, manufacturing of flexible graphene devices.

Conventional microfabrication-based photolithography requires extensive process steps, and yet suffers from polymeric residue^28^ introduced during graphene patterning. Furthermore, while graphene has the potential for novel applications such as flexible circuits and wearable electronics, the intended plastic substrates are not likely to be compatible with the organic solvents used in photolithography. In addition, the cost and lead-time associated with the fabrication of photolithography masks could be prohibitive in an iterative design process. On the other hand, transfer printing (or soft lithography) is constrained by the graphene source (e.g., graphene oxide), transfer layer, and target substrate^17^,^18^. Intricate photolithography-based stamp fabrication for the transfer layer and non-uniformity of transferred graphene layers are limitations of this technique. Direct patterning based on optical drive enabled laser scribing shows promise in commercial applications^20^, but the process is only compatible with direct laser reduction of graphene oxide films, and is constrained to serial processing with limited scalability. While nanometer-scale patterning of single layer graphene through laser ablation has been reported^21^, the process requires an expensive femtosecond laser. Finally, direct growth of patterned graphene requires the catalyst to be patterned before the growth step by the same extensive processing infrastructure as mask photolithography and/or is limited to high temperature resistance surfaces^14^,^23^,^24^,^25^,^26^,^27^. Furthermore, attempts to achieve one-step catalyst-free direct growth of patterned graphene on a target substrate reportedly yield subpar quality graphene^23^.

Here we report a one-step facile method to pattern graphene by using stencil mask and oxygen plasma reactive-ion etching (RIE), and subsequent polymer-free direct transfer to flexible substrates. In conjunction with the recent evolution of additive and subtractive manufacturing techniques such as three-dimensional (3D) printing and computer numerical control (CNC) milling, we developed a simple and scalable graphene patterning technique using a stencil mask fabricated via a laser cutter. Our approach to patterning graphene using a stencil mask is based upon the shadow mask technique that has been employed for contact metal deposition^29^,^30^. Not only are these stencil masks easily and rapidly manufacturable (for iterative rapid prototyping), but they are also reusable, enabling cost-effective pattern replication. Furthermore, since our approach involves neither a polymeric transfer layer nor chemicals (organic solvents), we are able to obtain contamination-free graphene patterns directly on various flexible substrates.

## Results and Discussion

**Figure 1:**
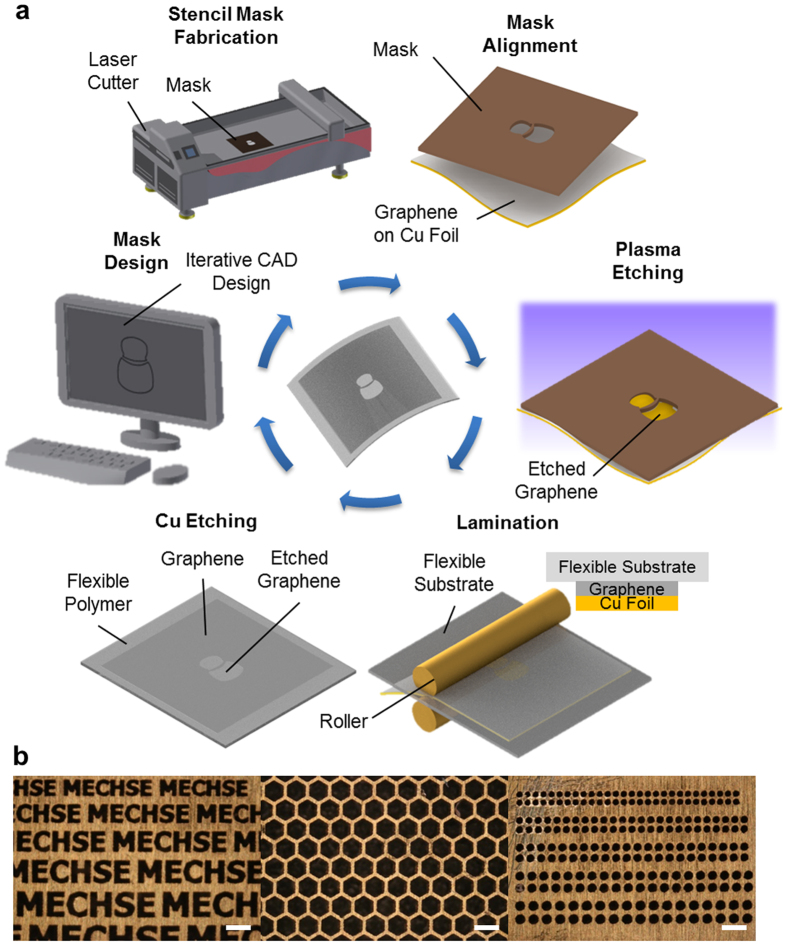
One-step graphene patterning and polymer-free graphene transfer onto a flexible substrate. (**a**) Detailed schematic illustration of the one-step polymer-free approach to fabricate patterned graphene on a flexible substrate. A stencil mask is designed by a CAD software and fabricated by a laser cutter. The fabricated mask is aligned on the as-grown CVD graphene on a Cu foil, and the exposed graphene region is removed by oxygen plasma. The patterned graphene is laminated onto a flexible substrate, followed by etching of the Cu foil. (**b**) Optical microscope images of various stencil masks with sophisticated micro-scale features. All scale bars: 300 μm.

In Fig. 1, we illustrate our one-step and polymer-free technique to pattern CVD grown graphene and transfer the patterned graphene onto a flexible substrate. We used a commercially available laser cutter (40 μm spot size) to fabricate stencil masks. Desired micropatterns are designed by a computer-aided design (CAD) software, and the laser cutter interfaced to the computer fabricated a stencil mask accordingly. The rapid production of stencil mask is facilitated by the quick turnover of the laser cutter to pattern low cost polymer and metal sheets (See Supplementary Methods). As examples, brass and Kapton stencil masks with various features are shown in Fig. 1b and Fig. S1. The fabricated mask is first aligned on the as-grown CVD graphene on a copper foil and then patterned by O_2_ plasma etching. While oxygen gas is commonly used for plasma etching of graphene^31^,^32^,^33^,^34^, there have been several studies on other promising gases, such as argon^31^ and hydrogen^35^ for plasma etching of graphene. Subsequently, the patterned graphene is transferred onto a flexible substrate through a lamination process to ensure conformal contact, followed by removal of Cu foil by etching process (See also Methods). Due to this facile approach, our method reduces the total fabrication steps and time by eliminating the need for an intricate microfabrication process (and the need for a polymeric scaffold). More importantly, our polymer-free approach promotes cleaner graphene.

**Figure 2:**
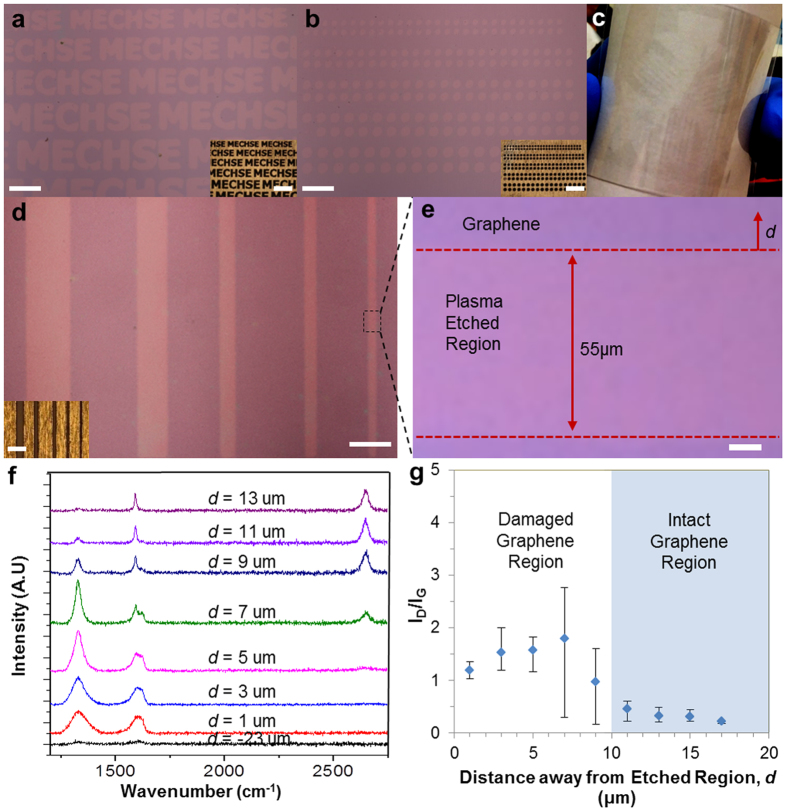
Characterization of patterned graphene features. (**a**,**b**) Optical microscope images demonstrating various graphene array patterns of different sizes transferred onto a SiO_2_ substrate. Insets show stencil masks used for graphene patterning. Scale bars: 300 μm (main) and 600 μm (inset). (**c**) Photograph showing that patterned graphene is successfully transferred onto a flexible substrate, PET. (**d**) Optical microscope image of a line pattern array showing the region where graphene quality is assessed. Scale bar: 300 μm (main) and 600 μm (inset). (**e**) Optical microscope image (rotated by 90°) of the line pattern delineated by the dotted region in (**d**). Scale bar: 10 μm. (**f**) Respective Raman spectrum from plasma etched region by varying distance, *d*. (**g**) Characterization of the intact and damaged graphene regions of patterned graphene prepared by our approach. Graphene in the region with a distance of ~10 μm from the patterned edge exhibits good quality.

We demonstrate the potential of our method for effective pattern replication and parallel processing in device fabrication. To show such potential, we produced micron-sized features with a variety of repeated shapes etched on the graphene sheet grown on copper, and then successfully transferred the patterned graphene onto rigid and flexible substrates in a conformal fashion. Optical microscope images (Figs 2a,b,d, and S2) demonstrate various graphene array patterns with different sizes, including lines, letters, and circles of ~50 μm in size. The graphene array patterns were transferred on a SiO_2_/Si substrate with 300 nm thermal oxide for better visual contrast. Figure 2c shows our capability to transfer patterned graphene onto flexible polyethylene terephthalate (PET) substrate. Additional photographs of flexible substrates such as Kapton film and polydimethylsiloxane (PDMS) with patterned graphene are provided in Fig. S3. A multitude of such patterns demonstrates the versatility of our patterning method by stencil masks and compatibility with various substrate choices.

To characterize the pattern replication capability and resolution of our stencil-based patterning, we determine the ratio of feature size of patterned graphene to that of stencil mask for various design feature sizes (Fig. S4). Unity (represented by dotted line) in the figure indicates that there is a perfect size match between the hollow feature of the mask and that of the patterned graphene. The mismatch in size between patterned graphene to mask was between 2 to 12% (conformity ratio of 1.02 to 1.12) for feature sizes in the range of 50–300 μm (Fig. S4). This mismatch is attributed to a small inherent gap between the flexible mask and graphene, leading to plasma leakage during the etching process. We note that the mechanical flexibility of our metal thin film or Kapton mask is a strength for pattern replication as the flexible mask can potentially conform better to graphene on flexible copper foil.

To substantiate the quality and integrity of our patterned graphene, we carried out Raman analysis at different distances from the etched feature edge. Figure 2d,e show the optical microscope image of a patterned array with delineated region where graphene quality was assessed. Figure 2f illustrates the respective Raman spectrum at different locations in Fig. 2e (denoted by distance d from the feature edge). First, we observed that graphene successfully etched in the center zone (d = −23 μm) shows no apparent peaks distinctive to graphene within the specified wavenumber range (1200–3000 cm^−1^)^36^ . Second, since there is an inherent gap between graphene and stencil mask, patterned graphene close to the edge exhibited defects caused by the leakage of O_2_ plasma (for d = 1 to 9 μm)^32^,^37^,^38^. We used the Raman D-to-G peak intensity ratio to systematically characterize the integrity of graphene (Fig. 2g)^36^; low ratio (<~0.3) indicates an intact graphene^38^, while higher ratio indicates a damaged graphene. Based on the investigation, we conclude that the quality of patterned graphene remains intact excluding the region within d = ~10 μm from the etched feature edge (Fig. 2f,g). We note that optimization of oxygen plasma conditions can be tailored to further reduce the defective zone^38^.

**Figure 3:**
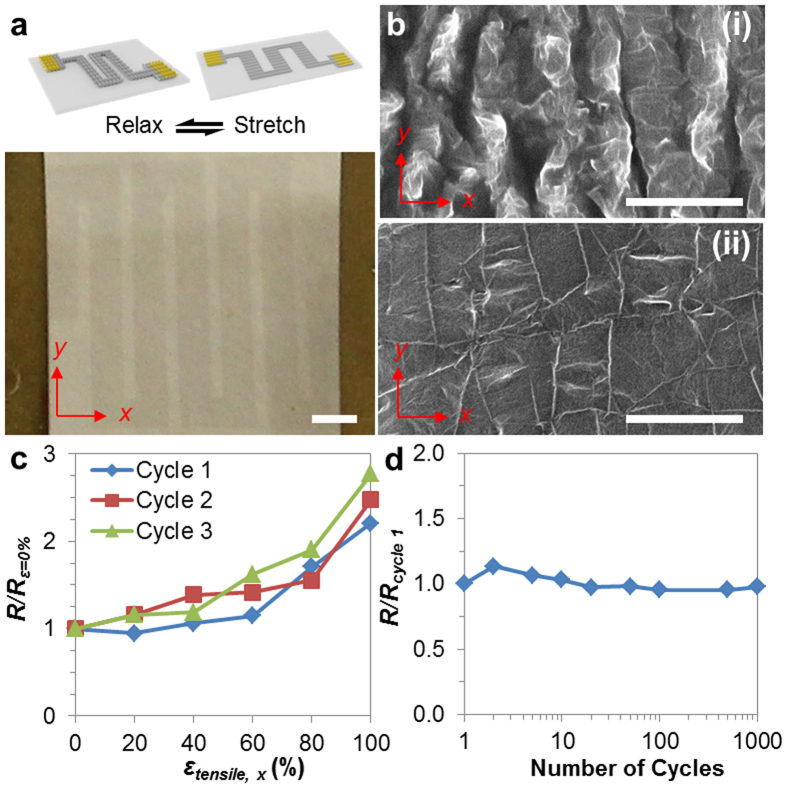
Rapid prototyping of stretchable graphene strain sensor. (**a**) Photograph of a crumpled graphene strain sensor device on a VHB film. Scale bar: 100 μm. Inset at the top shows the mechanism of sensing strains in *x*-direction. (**b**) SEM images of crumpled graphene structures (i) under relaxed and (ii) stretched state in *x*-direction. The VHB pre-stretched strains in *x*- and *y*- directions are ~300% and ~150%, respectively before being relaxed. The re-stretched strain is ~150% in *x*-direction. Scale bar: 5 μm. (**c**) Normalized change in resistance with varying strain from 0% to 100% over three cycles. (**d**) Change in resistance normalized with the resistance after 1^st^ cycle over one thousand cycles of stretching strains.

To investigate the capability of rapid prototyping for graphene-based functional devices, we demonstrate a stretchable, serpentine-shaped, crumpled graphene strain sensor fabricated by our stencil-based patterning/lamination approach. We patterned the graphene into a serpentine-shape pattern and subsequently generated out-of-plane crumpled structures by strain-induced buckle-delamination process (Fig. 3a)^39^,^40^. More specifically, patterned graphene was laminated onto a pre-strained elastomeric substrate followed by a subsequent release of the pre-strain to induce crumpling of graphene^41^. We applied a biaxial pre-strains of *ε*_*pre,x*_ ~ 250% and *ε*_*pre,y*_ ~ 150% before the patterned graphene transfer (See Methods). Scanning electron microscope (SEM) images (Fig. 3b) show the development of graphene crumple patterns under relaxed and stretched state^39^,^40^,^42^. As the graphene device is stretched uniaxially, there is an observable de-crumpling in the stretch direction.

To demonstrate the piezoresistive sensing capability of our strain sensor, we performed systematic studies of resistance under uniaxial tensile strain. The resistance of serpentine-shaped, crumpled graphene structure was measured at varying uniaxial tensile strains from 0% to 100% and the change in resistance compared to that at *ε*_*tensile,x*_ = 0% was plotted over applied strain (Fig. 3c). Increase in normalized resistance was observed with higher applied strain. The change in normalized resistance (Δ*R*/*R*_*0*_) versus increasing tensile strains (*ε*_*tensile,x*_) exhibited two different regimes^43^ (Figs 3c and S5): at lower strain, the strain sensitivity (gauge factor) is ~0.46 (*ε*_*tensile,x*_ = 0 to 40%), while at higher strain, the strain sensitivity is ~2.52 (*ε*_*tensile,x*_ = 40 to 100%) (Fig. S5). This compares favorably with the earlier rectangular-shaped, crumpled graphene strain sensor with a gauge factor of ~0.55^44^.

Furthermore, to demonstrate the robustness of our serpentine-shaped, crumpled graphene strain sensor, we characterized our device under cyclic strains. We measured the change in resistance with respect to the resistance at 1^st^ cycle (*R*/*R*_*cycle 1*_) over one thousand cyclical strains of *ε*_*tensile,x*_ ~ 50% (Fig. 3d). Two terminal resistance measurements showed no significant increase under cyclic strain, indicating that the graphene integrity was preserved owing to its reversible crumpling/de-crumpling process of serpentine-patterned graphene obtained by our process (Fig. 3d)^41^.

Graphene patterning can also have significant impact in areas that require selective hydrophobicity or combined hydrophilic-hydrophobic patterns^45^. Graphene coated surfaces (*e.g.*, condenser pipes) with hydrophobic characteristics can enable dropwise condensation and therefore improve heat transfer characteristics. Recently, researchers have shown that hydrophobic and hydrophilic channels have improved condensation heat transfer performance compared to only hydrophobic surfaces based on the size and shape of hydrophilic region^46^. Hydrophilic-hydrophobic patterns are also promising for shaping and positioning of droplets for sequencing and sensing, liquid microfluidics, electrowetting display and surface tension driven microfluidics^47^,^48^.

**Figure 4:**
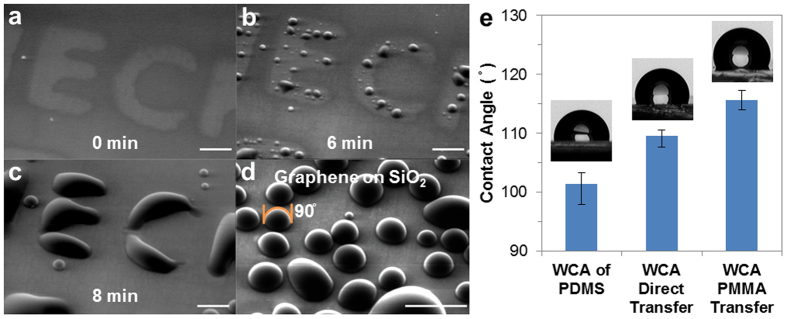
Rapid prototyping of patterned graphene condensation channels. (**a**–**c**) Time sequential E-SEM images of filmwise condensation on a bare SiO_2_ substrate in the form of letters “E” and “C”. Droplet condensation is observed at graphene region outside of the letter “E” and “C”. Scale bar: 50 μm. (**d**) E-SEM image of dropwise condensation on graphene portion of the sample shown in (**c**). Scale bar: 50 μm. (**e**) Water contact angle measurements to investigate the graphene surface cleanness: left (PDMS control), middle (graphene by our polymer-free transfer approach), right (graphene exposed to PMMA). Inset shows side view photographs of a water droplet on respective substrates.

We investigated the condensation performance of one-step patterned graphene features with an environmental SEM (E-SEM). The E-SEM allows real-time image acquisition during water vapor condensation on a cooled sample. Our capability to selectively pattern graphene resulted in filmwise condensation on the bare SiO_2_ substrate, as clearly shown by water film having the shape of letter “E” and “C” (Fig. 4a–c), in contrast to dropwise condensation on graphene surfaces elsewhere (Fig. 4c,d) (See also Supplementary Movie 1). In addition, water contact angle (WCA) is 90° at ~10 μm from the pattern edge. This WCA value matched with the value in the literature for single layer graphene^5^, demonstrating graphene was not damaged by the patterning process (at ~10 μm distance from the edge of the patterned feature). We note that the results herein are consistent with our findings from Raman spectroscopy (Fig. 2g).

To establish that our polymer-free process yields graphene devices with cleaner surface characteristics, macroscopic WCA measurements were carried out using a goniometer (Fig. 4e). The graphene surface that has more polymeric contaminants^28^ tends to show more hydrophobic behavior or higher WCA^49^. Graphene transferred by our polymer-free approach was compared with a graphene sample that was transferred by poly-methyl methacrylate (PMMA)-assisted conventional transfer approach (PMMA layer was removed by acetone before measurement; See Supplementary Methods). There was a 10° increase in WCA for the graphene sample on PDMS that was exposed to PMMA. These results show that our polymer-free approach produces cleaner graphene surfaces and promotes higher quality graphene.

Our approach demonstrates a new possibility to overcome limitations imposed by existing post-synthesis processes to achieve graphene micro-patterning. Our method to pattern graphene with a stencil mask followed by direct lamination transfer has several key advantages over existing techniques. First, the simple patterning process circumvents the costly lithographic patterning and post processing steps required in traditional patterning techniques. Furthermore, our method allows rapid design iterations and pattern replications. Second, our proposed polymer-free patterning technique promotes graphene of cleaner quality than fabrication techniques that necessitate graphene exposure to resist or PMMA. Third, the ubiquity, scalability, and low cost of stencil mask fabrication by additive and subtractive manufacturing techniques such as laser cutter and 3D printing offers a pathway that is conducive to discovering potential applications. Lastly, our patterning technique provides a simple and robust platform that can be applicable to patterning of a wide range of other novel two-dimensional materials of recent interest (*e.g.*, hBN, MoS_2_ and other transition metal dichalcogenides)^50^,^51^. Nevertheless, our method has some present limitations that requires further refinement. Currently, our patterning technique is limited to micron-sized graphene patterns with a micrometer scale defect zone. However, this resolution is limited by stencil mask fabrication tools (*e.g.*, laser cutter and 3D printer) rather than the process itself. Furthermore, previous studies have shown enhanced oxidation of graphene by O_2_ plasma in a slightly reducing environment of ammonia and argon (or hydrogen) for controlled etch rate predominantly at the graphene nanoribbon edges^52^.

## Conclusions

In conclusion, we have demonstrated a one-step, facile and scalable patterning of graphene using a stencil mask. We have shown that graphene can be patterned into varying geometrical shapes and sizes. Furthermore, we have explored various substrates for the direct transfer of the patterned graphene. Our approach produces cleaner graphene compared to fabrication techniques that utilize resist and polymeric scaffold for the transfer onto desired substrates. Finally, we have demonstrated the rapid prototyping capability of our technique to fabricate a stretchable, crumpled graphene strain sensor and condensation channels for potential heat management applications.

## Methods

### Sample Preparation

Graphene was grown on a copper foil by low-pressure CVD using a mixture of methane (CH_4_), hydrogen (H_2_), and argon (Ar). Subsequent graphene patterning on as grown CVD-grown graphene was achieved after by aligning stencil mask first, then by oxygen plasma reactive-ion etching. The stencil masks shown in Fig. 1 were fabricated by a laser cutter (Potamac, MD). The transfer of patterned graphene onto a polymer substrate was attained by a commercial laminator (300-SCL DSB, China) to ensure conformity at room temperature. The transfer was then followed by the etching of Cu foil by sodium persulfate solution. The detailed procedures and conditions during sample preparation, e.g. stencil mask fabrication, graphene synthesis, and sample preparation for water contact angle measurements, are provided in Supplementary Information.

### Characterization

Optical microscope images were captured in a reflective bright-field mode (Axio Imager M2m, Carl Zeiss, Germany). E-SEM images (at 4 °C sample temperature, and 100% relative humidity) were obtained using FEI quanta 450 SEM (FEI, OR) by increasing water vapor pressure to or above saturation pressure during E-SEM imaging. Raman spectra were obtained with the laser excitation wavelength of 633 nm, 1800 l/mm grating, 10 seconds of accumulation (Renishaw Raman/PL Micro-spectroscopy, Gloucestershire, UK). Water contact angles were measured by a goniometer KSV CAM200 (KSV Instruments Ltd., Helsinki, Finland).

### Fabrication/Characterization of Patterned, Crumpled Graphene Strain Sensor

A VHB tape (3M, MN), a highly stretchable acrylic film, was biaxially pre-strained by *ε*_*pre,x*_ ~ 250% and *ε*_*pre,y*_ ~ 150%. Patterned, serpentine-shaped graphene on a Cu foil was then transferred onto a pre-strained VHB film. The Cu foil was then chemically etched with sodium persulfate solution. Thin gold film (40 nm) was deposited by thermal evaporator (Nano 36, Kurt J. Lesker, PA) to create contact pads. The device was biaxially re-stretched during the thermal deposition to create corrugated gold contact electrodes serving the device stretchability. The crumpling of graphene serpentine structures was accomplished by subsequent releasing of the elastomeric substrate.

Two terminal resistance measurements were performed with a probe station (PM8, SUSS Micro Tec, Germany) and a sourcemeter (2614B, Keithley Instruments, OH). The serpentine-shape crumpled graphene strain sensor device was uniaxially stretched along the direction with higher pre-strain (*x*-direction as illustrated in Fig. 3a). We monitored the conductance of the device at varying tensile strains.

## Additional Information

**How to cite this article**: Yong, K. *et al.* Rapid Stencil Mask Fabrication Enabled One-Step Polymer-Free Graphene Patterning and Direct Transfer for Flexible Graphene Devices. *Sci. Rep.* **6**, 24890; doi: 10.1038/srep24890 (2016).

## Supplementary Material

Supplementary Information

Supplementary Movie 1
