# Supplementary material for: Rapid Stencil Mask Fabrication Enabled One-Step Polymer-Free Graphene Patterning and Direct Transfer for Flexible Graphene Devices
Source: Sci Rep. 2016 Apr 27;6:24890. doi: 10.1038/srep24890 (PMC4846816; doi:10.1038/srep24890)
Supplement: Supplementary Information [file srep24890-s1.pdf]

Supplementary Information

Rapid Stencil Mask Fabrication Enabled One-Step Polymer-Free Graphene Patterning and Direct Transfer for Flexible Graphene Devices

Keong Yong[1,†], Ali Ashraf[1,†], Pilgyu Kang[1], and SungWoo Nam[*,1,2]

[1]Department of Mechanical Science and Engineering and [2]Department of Materials Science and Engineering, University of Illinois at Urbana−Champaign, Urbana, Illinois 61801, United States

**Corresponding Author:* swnam@illinois.edu

† *These authors contributed equally to this work.*

Supplementary Methods

Supplementary Figures

Supplementary Movie

Supplementary Methods

Stencil Mask Fabrication

The brass stencil mask (0.005” thick) shown in Figure 1b was fabricated by a laser cutter (Potomac, MD). For our range of power settings (50 W), brass film yielded the best and most consistent spatial resolution among the mask materials tested (including brass film, stainless steel film, Kapton film, *etc.*). Certain mask materials such as Kapton film would burn or warp if the laser micromachined beyond its optimal process settings (power, speed, and number of passes). Hence, each mask material requires careful adjustment of the process settings. Importantly, laser cutting is not the only technique available for stencil mask fabrication. Other additive or subtractive manufacturing techniques include three-dimensional (3D) printing and computer numerical control (CNC) milling.

Graphene Synthesis

Graphene was synthesized on a 25 μm thick copper (Cu) foil (Alfa Aesar, MA) by chemical vapor deposition (CVD) system (Rocky Mountain Vacuum Tech Inc., CO) with methane (CH_4), hydrogen (H_2), and argon (Ar) as gaseous precursors. Cu foil was pretreated with concentrated hydrochloric acid (HCl) (Sigma Aldrich, MA) for 10 minutes prior to synthesis. After loading the catalyst substrate (Cu foil) at the center of the quartz CVD chamber, the temperature of chamber was raised to 1050 °C under the flow of H_2 (50 sccm) at 150 mTorr. Annealing was performed at 1050 °C for 35 minutes. Then CH_4 (10 sccm) was introduced into the chamber along with H_2 at 520 mTorr for 10 minutes for graphene synthesis. Finally, the chamber was cooled down slowly while fast cooling was applied to the Cu foil using a loadlock under Ar flow (500 sccm).

Graphene Patterning and Transfer

We perform one-step graphene patterning and polymer-free graphene transfer on a flexible substrate as illustrated in Figure 1a according to the following steps: The fabricated stencil mask was first aligned on the as-grown CVD graphene on a Cu foil. Next, uniform pressure was applied through large glass slides to ensure conformal contact. Then, graphene patterning was achieved by oxygen plasma reactive-ion etching (Diener GmbH, Germany) for 30 seconds (500 W & 150 mTorr). Graphene on Cu foil was then transferred onto a polymer substrate using a commercial laminator, 300-SCL (DSB, China), at room temperature to ensure conformity. The lamination configuration is illustrated in Figure 1a. The Cu foil was then chemically etched by sodium persulfate solution ($Na_2S_2O_8$) (Sigma Aldrich, MA). The transfer was completed by rinsing the sample in multiple water baths. The patterned graphene on SiO_2 substrates as shown in Figure 2a, 2b, 2d, and S2 are transferred by the conventional method using poly-methyl methacrylate (PMMA) (Sigma Aldrich, MD) as a scaffold (dissolved after transfer).

Water Contact Angle Measurements

Water contact angles (WCA) were measured by a goniometer KSV CAM200 (KSV Instruments Ltd., Helsinki, Finland). To demonstrate that our polymer-free process yields graphene devices with a cleaner surface (Figure 4e) than the conventional PMMA-assisted transfer method, the WCA of graphene under different treatments were obtained by a goniometer in a Class 100 cleanroom. Deionized water (18.2 MΩ•cm) was used for WCA measurements. The WCA of polydimethylsiloxane (PDMS) (Dow Corning, MI) was measured as-is to serve as a control (1st sample). The graphene was transferred onto the PDMS by our polymer-free

approach (2nd sample). The other graphene sample was transferred onto the PDMS with an exposure to PMMA, which was later dissolved in acetone (3rd sample).

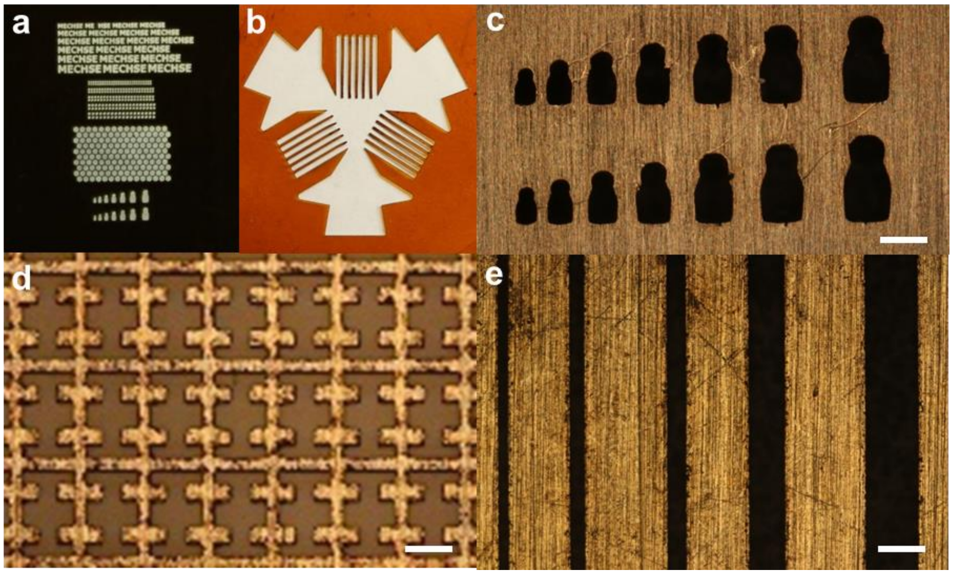

Figure S1. (a-b) Photographs of brass stencil mask and Kapton mask. (c-e) Optical microscope images of various brass mask features to highlight the ease of translating a computer-aided design to an actual stencil mask. All scale bars: 300 µm.

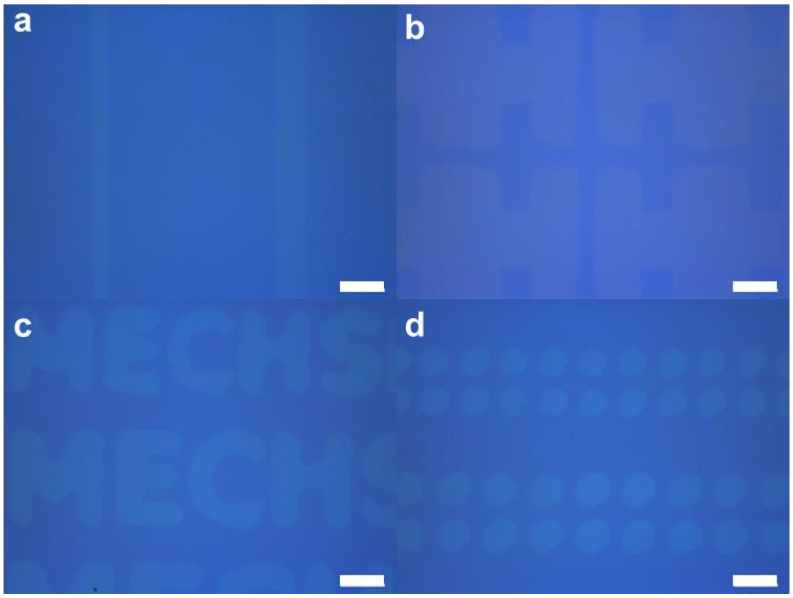

Figure S2. Optical microscope images of graphene patterns on a SiO_2 substrate with 20X objective lens. Smallest features are *ca.* 50 µm, compared to laser spot size of *ca.* 40 µm. All scale bars: 100 µm.

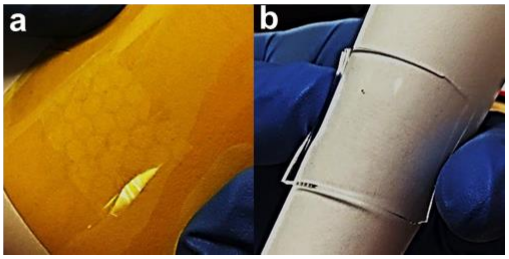

Figure S3. Photographs showing that patterned graphene is successfully transferred onto various flexible substrates: (a) Kapton and (b) PDMS.

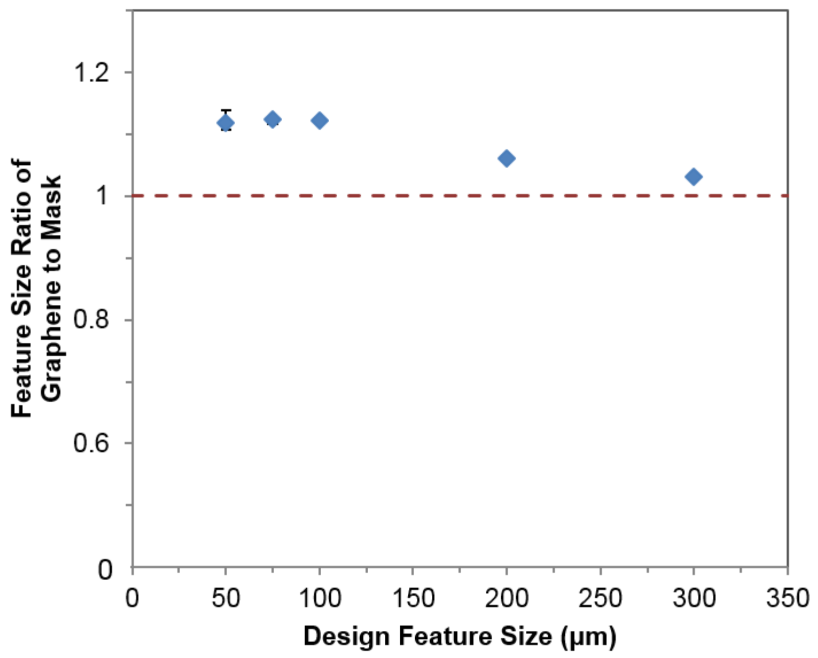

Figure S4. Feature size ratio of patterned graphene on SiO_2 to that of the stencil mask. The size ratio for the value of unity is denoted by a dotted red line indicating the matching of obtained feature with designed feature.

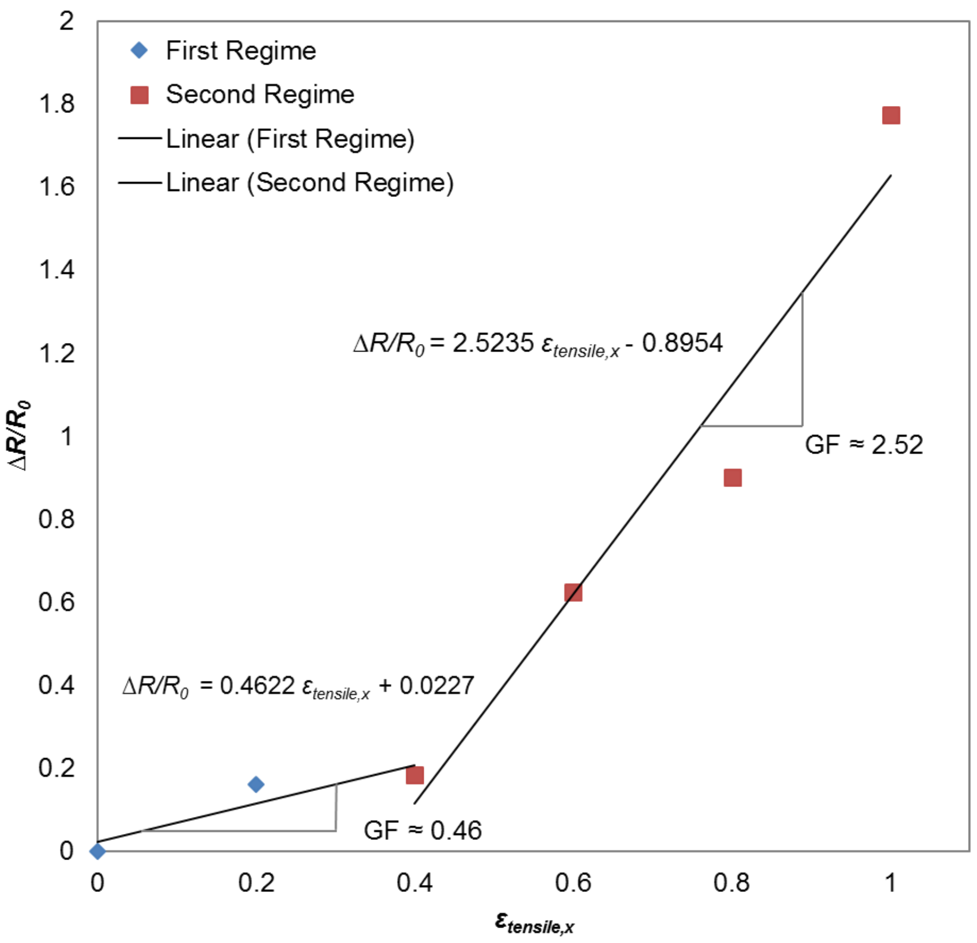

Figure S5. Gauge factor (GF) characterizations for our serpentine-patterned, crumpled graphene strain sensor. Gauge factor is a measure of strain sensitivity as follows: GF = $(\Delta R/R_0)/\varepsilon_{tensile,x}$, where R is the resistance and ε is the strain applied. Continuous increase in normalized resistance was observed with increasing applied strain. There are two different regimes: (i) lower strain sensitivity (gauge factor) at lower strain value ($\varepsilon_{tensile,x} < 0.4$), and (ii) higher strain sensitivity at higher strain value ($0.4 < \varepsilon_{tensile,x} < 1$). The serpentine-patterned, crumpled graphene strain sensor yielded gauge factors of ~0.46 for the first regime and ~2.52 for the second regime.

Supplementary Movie. Water condensation time lapse captured by environmental scanning electron microscopy. The etched regions (letters “S” and “H”; SiO_2 substrate) are marked by a pink color at the beginning. The video shows dropwise condensation on graphene over time, in contrast to filmwise condensation on a SiO_2 substrate.
